# A novel tumor suppressor ASMTL-AS1 regulates the miR-1228-3p/SOX17/β-catenin axis in triple‐negative breast cancer

**DOI:** 10.1186/s13000-021-01105-3

**Published:** 2021-05-18

**Authors:** Jie Sun, Xiaohua Li, Enqiao Yu, Jianxia Liu, Liang Sun, Qin He, Qiran Lu

**Affiliations:** 1 Department of Breast Surgery, The 1st Affiliated Hospital of Soochow University, Ward 6, 2F Surgical Building, 188 Shizi Street, Gusu District, Jiangsu 215006 Suzhou, China; 2 Department of Breast Surgery, Wuzhong People’s Hospital, 215128 Jiangsu Suzhou, China; 3 Department of Breast Surgery, Dushuhu Public Hospital Affiliated to Soochow University, 215006 SuZhou, JiangSu China

**Keywords:** ASMTL-AS1, TNBC, Wnt/β-catenin pathway, miRNA sponges, Prognosis

## Abstract

**Background:**

Triple-negative breast cancer (TNBC) is a special type of breast cancer that lacks effective therapeutic targets. There is a significant need to clarify its pathogenesis, so as to bring new targeted approaches for TNBC management. Here, we identified a long-non coding RNA (lncRNA) ASMTL-AS1 that linked to TNBC development and progression.

**Methods:**

Quantitative real-time polymerase chain reaction (qRT-PCR) and Western blot assays were used to test gene and protein levels, respectively. The regulatory axis of miR-1228-3p/SOX17/β-catenin was determined by luciferase reporter and RNA pull-down assays. *In vivo* assay was conducted by using the nude mice model via subcutaneous transplantation of tumor cells.

**Results:**

ASMTL-AS1 was significantly downregulated in TNBC tissues compared to normal tissues, which was closely associated with aggressive clinical features and unfavorable prognosis. Lentivirus-mediated ASMTL-AS1 overexpression evidently reduced the ability of TNBC cell colony formation, activity and invasion by more than 2.5 times. RNA pull-down and luciferase reporter assays revealed that miR-1228-3p directly bound to ASMTL-AS1, ASMTL-AS1 increased SOX17 expression via sponging and repressing miR-1228-3p. Subsequently, the upregulated SOX17 trans-suppressed β-catenin expression, resulting in the inactivation of carcinogenic Wnt/β-catenin signaling, thereby restraining TNBC cell growth and dissemination. Importantly, the xenograft tumor model showed that the ASMTL-AS1 overexpression significantly retarded tumor growth, and negatively regulated Wnt/β-catenin pathway.

**Conclusions:**

Our data characterize a novel tumor suppressor in TNBC, restoration of ASMTL-AS1 may be a candidate therapeutic intervention for TNBC patients.

## Background

Breast cancer has now overtaken lung cancer as the leading cause of cancer incidence worldwide regardless of gender, with an estimated 2.3 million new cases, accounting for 11.7 % of all cancer cases[1]. As a specific subtype of breast cancer, triple-negative breast cancer (TNBC) is characterized by negative estrogen receptor (ER), progesterone receptor (PR) and human epidermal growth factor receptor 2 (HER-2), accounting for about 15 ~ 20 % of all breast cancer cases[2]. Chemotherapy is the main treatment for TNBC, such as paclitaxel and platinum drugs and so on, while targeted and endocrine therapy are basically ineffective[3]. However, chemotherapy drugs have no selectivity, while killing cancer cells, they will also cause damage to normal cells, causing some adverse reactions[4]. Therefore, in-depth study of the etiopathogenesis of TNBC in order to find effective therapeutic targets has become a research hotspot in the current medical field.

Long non-coding RNA (lncRNA) is a kind of endogenous RNA with transcripts longer than 200nt, which was previously thought to be the “noise” of transcriptome, with no definite biological function[5]. In recent years, with the deepening of research, more and more evidence shows that lncRNA participates in individual growth, development and cell differentiation, and acts as a crucial regulator of gene expression at the transcriptional and post-transcriptional levels[6, 7]. LncRNA is frequently abnormally expressed in human cancer tissues, and is widely involved in many important regulatory processes, such as chromatin modification, transcriptional activation and interference and intranuclear transport, closely linking to cancer occurrence and development[8]. One of the main functions of lncRNA is adsorption of microRNAs (miRNAs), in which lncRNA competitively binds to miRNAs, relieving the suppressive effect of miRNA on target genes, this model is also known as “competing endogenous RNA (ceRNA)” network[9]. Extensive studies have shown that lncRNA serves as a ceRNA in various human diseases, such as the lnc-BCYRN1/miR-619-5p/CUEDC2 axis in glioma[10], lnc-DNM3OS/miR-126/IGF1 axis in osteoarthritis[11], and lnc-UCA1/miR-182-5p/DLL4 axis in renal cancer[12].

ASMTL-AS1 is a newly found lncRNA that locates at Xp22.33 and Yp11.2, which has been recently reported as a key player in papillary thyroid cancer[13] and hepatocellular carcinoma[14], and could be used as a prognostic biomarker for bladder cancer[15]. Nevertheless, its role in TNBC remains unknown. In this study, we aimed to explore the expression, function and clinical implication of ASMTL-AS1 in TNBC, furthermore, we also uncovered the tumor-inhibiting role of ASMTL-AS1 by acting as a ceRNA.

## Methods

### Tissue samples and cell lines and transfection

This study was approved by the medical ethics committee of The 1st Affiliated Hospital of Soochow University, which was conducted in strict accordance with the Helsinki Declaration. The matched TNBC and para-cancerous normal tissues were collected from 68 TNBC patients undergoing surgical resection at The 1st Affiliated Hospital of Soochow University. We obtained informed consent from all patients, and the clinicopathological features were displayed in Table 1. Two TNBC cell lines MDA-MB-231 and MDA-MB-468 were purchased from ATCC, cultured in RPMI-1640 medium, and stored in liquid nitrogen vapor phase at -80 °C. Cell transfection was performed using Lipofectamine 3000 (Invitrogen, CA, USA) according to supplier’s instructions. miRNA inhibitors, mimics and si-SOX17 (5`-GCAGAACCCAGATCTGCAC-3`) were purchased from Ribobio (Guangzhou, China).

**Table 1:** Association of ASMTL-AS1 expression with clinical features in 68 triple-negative breast cancer patients

Parameters	Total (*n* = 68)	ASMTL-AS1 expression		*P* value
		Low (*n* = 34)	High (*n* = 34)	
Age (years)				
≤ 40	21	9	12	0.431
> 40	47	25	22	
Menopause				
Yes	28	12	16	0.324
No	40	22	18	
Tumor size (cm)				
≤ 2	32	8	23	<0.001
> 2	36	25	11	
Lymph node metastasis				
Negative	25	7	18	0.006
Positive	43	27	16	
TNM stage				
I-II	23	2	21	<0.001
III	45	32	13	

### qRT-PCR analysis

Total RNA of tissues and cells was extracted by Trizol solution (Invitrogen), and RNA with different subcellular localization was separated by Cytoplasmic & Nuclear RNA Purification Kit as per supplier’s instructions (Norgen Biotek, ON, Canada). Then, the single-stranded cDNA was synthesized by GOScript™ reverse transcriptase (Promega, WI, USA), followed by quantification with GoTaq® qPCR Master Mix by 2^−ΔΔCt^ method. U6 and GAPDH were used as reference controls for nuclear and cytoplasmic fragments, respectively. The primers used in this study: ASMTL-AS1-Forward: 5`-TTGTGGACTTTGTCGTCTGG-3`, Reverse: 5`-CTGACGGACGTATCTCGTTTT-3`; Sox17-Forward: 5`-GGGGACATGAAGGTGAAGG-3`, Reverse: 5`-TTGTGCAGGTCTGGATTCTG-3`; CyclinD1-Forward: 5`-AACTACCTGGACCGCTTCCT-3`, Reverse: 5`-TCGGTGTAGATGCACAGCTT-3`; MMP7-Forward: 5`-AGCCAAACTCAAGGAGATGC-3`, Reverse: 5`-CCATTTTGGGCTATTTGGAA-3`; TWIST-Forward: 5`-AGCTGAGCAAGATCCAGACG-3`, Reverse: 5`-GGAGAAGGCGTAGCTGAGG-3`; β-catenin-Forward: 5`-ACTCGAGCTCAGAGGGTACG-3`, Reverse: 5`-TCTGTGATGGTTCAGCCAAA-3`.

### Generation of stable ASMTL-AS1-overexpressing cell lines

The full-length sequence of ASMTL-AS1 was synthesized and cloned into pLCDH-CMV-MCS-EF1-GFP-Puro lentiviral vector. Then, MDA-MB-231 and MDA-MB-468 cells were infected with above lentiviral vector in the presence of 5 µg/mL polybrene. Cells were routinely cultured in RPMI-1640 medium added with 1 µg/mL puromycin for 1 week. qRT-PCR analysis was applied to verify the infection efficiency.

### Colony formation and CCK-8 assays

500 MDA-MB-231 and MDA-MB-468 cells were seeded into 6-well plates, then routinely cultured in RPMI-1640 medium for 2 weeks. Cells were washed by PBS twice and fixed by methanol, the number of clones was observed under a light microscope. For testing cell viability, 1 × 10^4^ cells were seeded into 96-well plates and cultured for 72 h. 10µL CCK-8 reaction solution (Dojindo, Kumamoto, Japan) was added every 24 h and incubated at 37 °C for 1 h. The absorbance at 450nm was recorded by a microplate reader.

### Transwell assay

5 × 10^4^ MDA-MB-231 and MDA-MB-468 cells in 200µL RPMI-1640 medium were added into upper Boyden chambers (BD Inc., CA, USA) coated with matrigel, while 600µL RPMI-1640 medium was added into lower chambers. After incubation at 37 °C for 24 h, the cells on the lower surface of the filter were fixed, stained and examined using a light microscope.

### RNA pull‐down assay

For affinity pull down of endogenous ASMTL-AS1, the biotin-labeled DNA probe complementary to ASMTL-AS1 (5`-TCGTCCGGCCGGGGACTCCCTCTGTCGTCTC-3`) was synthesized (Sangon, Shanghai, China) and incubated with TNBC cell lysates for 3 h at 4 °C. Then, the Dynabeads M-280 Streptavidin beads (Invitrogen) was added into above cell lysates and incubated for 0.5 h at room temperature. RNA complexes were washed and eluted for qRT-PCR analysis of miRNA enrichment.

### Luciferase reporter assay

The sequences of ASMTL-AS1 and SOX17 3`-UTR containing miR-1228-3p binding site were cloned into the downstream of FL reporter vector (Obio, Shanghai, China). Then, the reporter vector was co-transfected with pRL-CMV Renilla luciferase reporter and miR-1228-3p mimics into TNBC cells using Lipofectamine 2000 (Invitrogen). After 48 h, the luciferase activity was tested by using the dual-luciferase reporter assay system (Promega).

### Western blot

Total protein was extracted by the mammalian protein extraction kit (CWBIO, Beijing, China) and quantified using the bicinchoninic acid (BCA) protein assay kit (CWBIO) following manufacturer’s instructions. Then, 10 µg protein was loaded into the SDS-PAGE gel and transferred onto PVDF membrane. After blocking with 5 % defatted milk powder for 30 min at room temperature, the membrane was incubated with primary and secondary antibodies. The protein signal was visualized using the Immobilon-Western HRP kit (Millipore) and exposed to X-ray films. The primary antibodies used in this study are as follows: anti-SOX17 (#81,778, CST), anti-SOX17 (#8480, CST) and anti-Tubulin (#2148, CST).

### Xenograft tumor experiment

A total of 6 nude mice were randomly divided into two groups, pLCDH-vector and pLCDH-ASMTL-AS1. 1 × 10^7^ control or ASMTL-AS1-overexpressing MDA-MB−231 cells were subcutaneously injected into nude mice. Tumor volume was measured every weeks by using a vernier caliper and calculated with length×width^2^/2 formula. After 5 weeks, tumor tissues were collected and weighted. Total RNA was extracted by Trizol solution, followed by qRT-PCR analysis.

### Data analysis

The average between the two groups was analyzed by Student’s t test, while Chi-square test was used to compare the categorical data, respectively. The survival curve was plotted by Kaplan-Meier method and analyzed by Log-rank test. *P* < 0.05 was set as statistically significant.

## Results

### ASMTL-AS1 is a downregulated lncRNA in TNBC

**Fig. 1:**
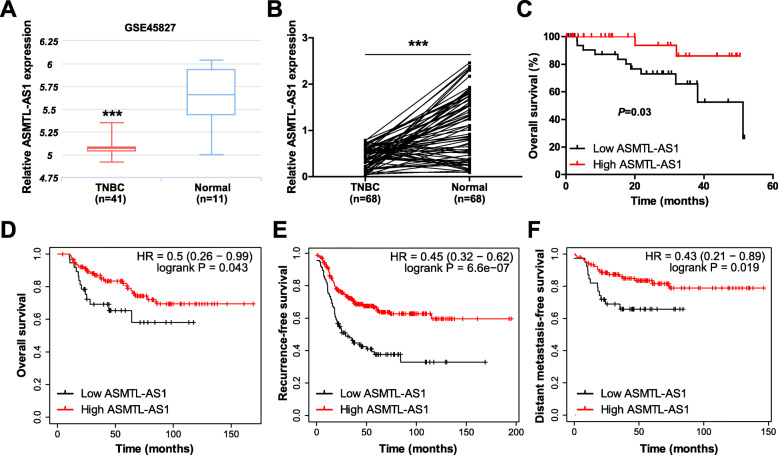
ASMTL-AS1 is significantly decreased in TNBC. **a** The expression of ASMTL-AS1 in GSE45827 database. **b** qRT-PCR analysis of ASMTL-AS1 expression in 68 matched TNBC and normal tissues. **c** The overall survival curve of TNBC patients with low and high ASMTL-AS1 levels in our cohort. **d**-**f**. The overall, recurrence- or distant metastasis-free survival curve of TNBC patients with low and high ASMTL-AS1 levels in Kaplan-Meier plotter database. ^***^P < 0.001. The high and low expression level of ASMTL-AS1 in this study was based on median ASMTL-AS1 level in TNBC tissues

First, we analyzed that expression of ASMTL-AS1 in GSE45827 GEO database containing 41 TNBC and 11 normal tissues, the results showed that ASMTL-AS1 was evidently decreased in TNBC tissues (Fig. 1 a). Then, we verified this downregulation in our cohort containing 68 pairs of TNBC and adjacent normal tissues (Fig. 1b). Further, the correlations between ASMTL-AS1 and clinical features of TNBC patients were analyzed. As shown in Table 1, low ASMTL-AS1 level was positively associated with larger tumor size, lymph node metastasis and advanced TNM stage, but not with age and menopause status. Importantly, patients with high ASMTL-AS1 had longer overall survival time than those with low ASMTL-AS1 (Fig. 1 c). Consistently, the survival data from Kaplan-Meier plotter online tool (http://kmplot.com/analysis/index.php?p=background) showed that ASMTL-AS1 expression was reversely correlated with overall, recurrence-free and distant metastasis-free survival rate (Fig. 1d-f). These data suggest that ASMTL-AS1 may be a protective lncRNA against TNBC.

### Exogenous overexpression of ASMTL-AS1 inhibits TNBC cell proliferation and invasion

**Fig. 2:**
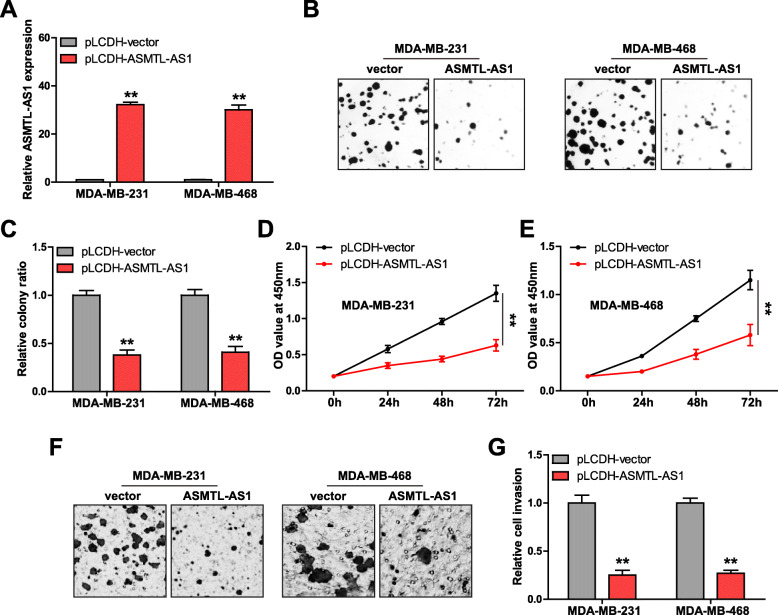
Overexpression of ASMTL-AS1 inhibits TNBC cell proliferation and invasion. **a**. qRT-PCR verifying the overexpression efficiency in two TNBC cells. **b**, **c**. The colony rate of TNBC cells after ASMTL-AS1 overexpression. **d**-**g**. CCK-8 and Transwell assays testing cell viability and invasion after ASMTL-AS1 overexpression, respectively. ^**^P < 0.01

We constructed two ASMTL-AS1-overexpressing TNBC cell lines via infection of lentiviral vector into MDA-MB-231 and MDA-MB-468 cells (Fig. 2 a). Then, a series of functional assays were carried out. As shown in Fig. 2b, c, ASMTL-AS1 overexpression resulted in a significant decreased in cell colony formation (average 59 versus 21). CCK-8 assay showed that cell viability was weakened in ASMTL-AS1-overexpressed MDA-MB-231 and MDA-MB-468 cells as compared to control cells (Fig. 2d, e). Likewise, Transwell assay showed that the number of invasive cells was substantially decreased after enforced expression of ASMTL-AS1 (average 25 versus 6) (Fig. 2 f, g).

### ASMTL-AS1 sponges miR-1228-3p in TNBC cells

**Fig. 3:**
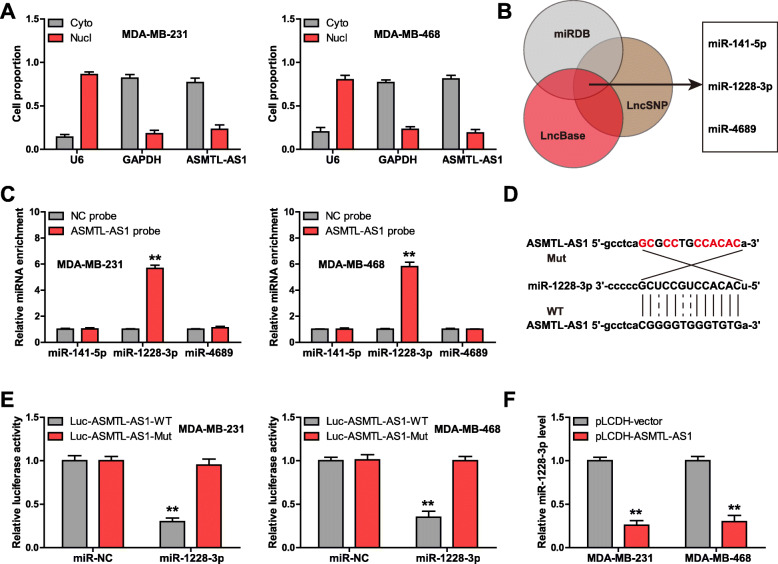
miR-1228-3p is a target of ASMTL-AS1. **a** qRT-PCR analysis of location of ASMTL-AS1 in MDA-MB-213 and MDA-MB-468 cells. **b** Three databases predicting miRNAs bound by ASMTL-AS1. **c** RNA pull-down assay using ASMTL-AS1 probe, followed by qRT-PCR analysis of miRNA enrichment. **d**, **e**. The binding site between ASMTL-AS1 and miR-1228-3p, followed by mutation and luciferase reporter assay. **f**. qRT-PCR analysis of miR-1228-3p level after ASMTL-AS1 overexpression. ^**^P < 0.01

Through subcellular localization analysis, we found that ASMTL-AS1 was dominantly located in the cytoplasm (Fig. 3 a), hinting that it may be a ceRNA sponging miRNAs. The intersection result of three online tools (miRDB: http://mirdb.org/index.html; LncBase: http://carolina.imis.athena-innovation.gr/; LncSNP: http://bioinfo.life.hust.edu.cn/lncRNASNP#!/) showed that there are three miRNAs (miR-141-5p, miR-1228-3p and miR-4689) sequences complementary to ASMTL-AS1 (Fig. 3b). We performed RNA pull-down assay to enrich endogenous miRNAs bound by ASMTL-AS1, the results showed that only miR-1228-3p was significantly pulled down by ASMTL-AS1 probe in both MDA-MB-231 and MDA-MB-468 cells (Fig. 3 c). Then, the binding site between ASMTL-AS1 and miR-1228-3p was mutated (Fig. 3d), followed by luciferase reporter assay. The results displayed that miR-1228-3p overexpression significantly reduced the luciferase activity of the wild-type reporter, but did not affect the mutant one (Fig. 3e). And miR-1228-3p expression was dramatically decreased in ASMTL-AS1-overexpressed TNBC cells in comparison to control cells (Fig. 3 f). These data indicate that ASMTL-AS1 is a ceRNA sponging miR-1228-3p in TNBC.

### ASMTL-AS1 represses Wnt/β-catenin signaling via the miR-1228-3p/SOX17 axis

**Fig. 4:**
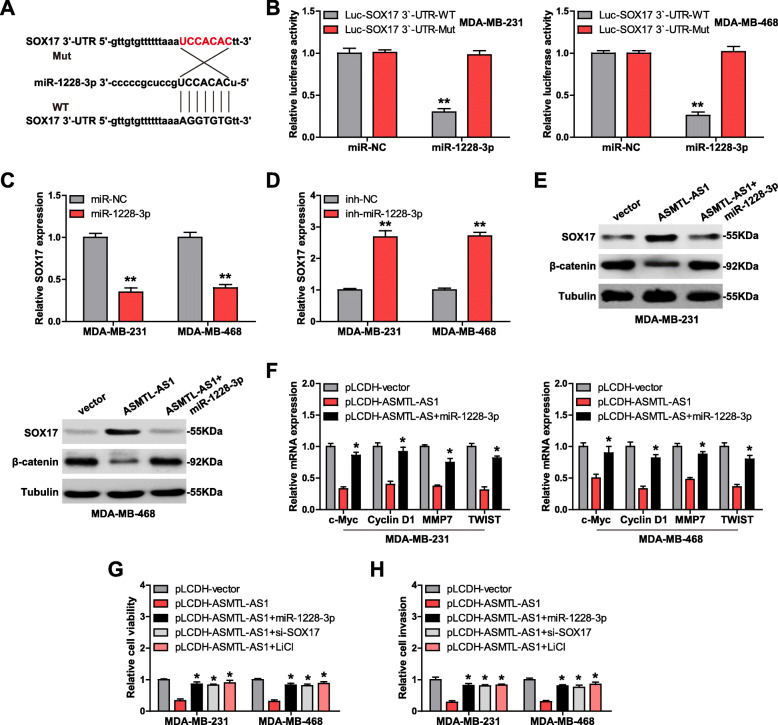
ASMTL-AS1 regulates the miR-1228-3p/SOX17/β-catenin axis. **a**, **b**. The binding site between SOX17 3`-UTR and miR-1228-3p, followed by mutation and luciferase reporter assay. **c**, **d**. qRT-PCR analysis of SOX17 mRNA level after alteration of miR-1228-3p expression. **e**. Western blotting testing SOX17 and β-catenin protein levels in ASMTL-AS1-overexpressing cells transfected with miR-1228-3p mimics. **f**. qRT-PCR analysis of β-catenin targets in ASMTL-AS1-overexpressing cells transfected with miR-1228-3p mimics. **g**, **h**. CCK-8 and Transwell assays respectively testing cell viability and invasion in ASMTL-AS1- overexpressing cells transfected with miR-1228-3p mimics, SOX17 siRNA or treated with LiCl, a specific activator of Wnt/β-catenin pathway. ^*^P < 0.05, ^**^P < 0.01

Through analyzing miRWalk online tool (http://zmf.umm.uni-heidelberg.de/apps/zmf/mirwalk2/) containing 8 prediction software, we found that SOX17, a transcription factor negatively regulating β-catenin expression, has the greatest possibility to be the downstream target gene of miR-1228-3p. We also mutated the complementary sequence between miR-1228-3p and SOX17 3`-UTR (Fig. 4 a), and performed luciferase reporter assay. Overexpression of miR-1228-3p reduced the luciferase activity of SOX17 3`-UTR vector, but this effect disappeared after mutation of the binding site (Fig. 4b). The expression of SOX17 was significantly reduced in miR-1228-3p-overexpressed cells (Fig. 4 c), while increased in miR-1228-3p-silenced cells (Fig. 4d). Importantly, ASMTL-AS1 overexpression led to a remarkable increase in SOX17 levels and subsequent decrease in β-catenin levels, whereas these effects were blocked by co-expression of miR-1228-3p (Fig. 4e). Furthermore, some well-known targets of β-catenin were also downregulated after ASMTL-AS1 overexpression, which was rescued by miR-1228-3p overexpression (Fig. 4 f). Functionally, the attenuated cell viability and invasion caused by ASMTL-AS1 overexpression were effectively antagonized by miR-1228-3p overexpression, SOX17 silencing or LiCl treatment (a specific activator of Wnt/β-catenin pathway) (Fig. 4g, h).

### ASMTL-AS1 overexpression retards TNBC cell proliferation ***in vivo***

**Fig. 5:**
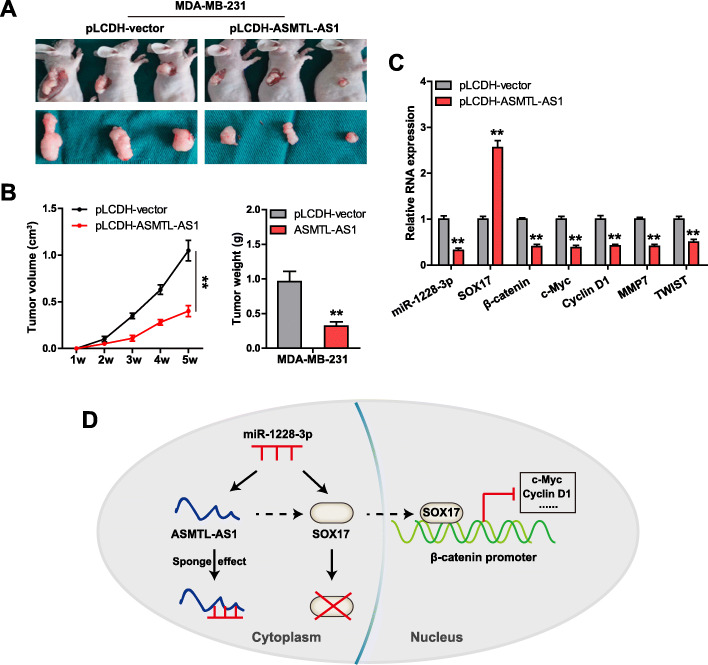
Recovery of ASMTL-AS1 inhibits tumor growth. **a**, **b**. Tumor volume and weight in control and ASMTL-AS1-overexpressing groups. **c**. qRT-PCR analysis of the indicated gene levels in control and ASMTL-AS1-overexpressing tumors. **d**. The mechanism diagram showing that ASMTL-AS1 inhibits TNBC progression via the miR-1228-3p/SOX17/Wnt/β-catenin axis

Lastly, we explored the *in vivo* function of ASMTL-AS1 using xenograft tumor model. As shown in Fig. 5 a, b, tumors induced by ASMTL-AS1-overexpressing MDA-MB-231 cells were significantly smaller than those developed from control cells. Further, qRT-PCR results showed that SXO17 was notably increased in ASMTL-AS1-overexpressing tumors, while miR-1228-3p, β-catenin, c-Myc, Cyclin D1, MMP7 and TWIST were significantly decreased (Fig. 5 c). These suggest that ASMTL-AS1 is a tumor-inhibiting lncRNA that regulates the miR-1228-3p/SOX17/β-catenin axis *in vivo*, which was in line with *in vitro* and clinical data.

## Discussion

Wnt/β-catenin signaling is a well-known oncogenic pathway and frequently abnormally activated in human cancer, including TNBC[16]. However, how it is activated in TNBC is still largely uncertain. In the present study, we found a lncRNA ASMTL-AS1 that negatively regulated Wnt/β-catenin activity via the miR-1228-3p/SOX17 axis. In detail, ASMTL-AS1 acted as a ceRNA that sponged miR-1228-3p and alleviated the binding effect of miR-1228-3p on SOX17 mRNA 3`-UTR, leading to upregulated SOX17, which bound to β-catenin promoter and inhibited β-catenin activation. Pre-clinically, we found that low ASMTL-AS1 was closely related to aggressive clinical features as well as dismal survival, and could be used as a promising prognostic biomarker for TNBC. Therefore, our data highlight the importance of ASMTL-AS1 in inhibiting TNBC development and progression, and also identity a novel regulator of Wnt/β-catenin signaling.

For disease research, biomarkers generally refer to a common physiological or pathological or a certain characteristic biochemical index in the treatment process that can be objectively measured and evaluated[17]. Through the determination of biomarkers, we can know the current biological process of the body. Detection of a disease-specific biomarker may be helpful for disease identification, early diagnosis and prevention, and monitoring in the process of treatment[18]. Thus, discovering valuable biomarkers has become a hot research topic. Very recently, a series of lncRNAs have been reported as cancer biomarkers, such as TP73-AS1 in glioma[19], BGL3 in papillary thyroid carcinoma[20], WT1-AS in lung adenocarcinoma[21], PHACTR2-AS1 in tongue squamous cell carcinoma[22], CRNDE in osteosarcoma[23] and LINC01451 in bladder cancer[24]. Here, we found that ASMTL-AS1 was significantly decreased in TNBC tissues, and patients with low ASMTL-AS1 had poor survival than patients with high ASMTL-AS1in both our cohort and Kaplan-Meier plotter data. These indicate that ASMTL-AS1 may be a candidate prognostic indicator for TNBC patients. Further studies involving large sample sizes are needed to validate its prognostic value in TNBC, and whether ASMTL-AS1 exists in body fluids, such as sweat, urine, blood or even exosomes, is worthy of further study, which may provide a non-invasive diagnostic biomarker for TNBC.

Recently, ASMTL-AS1 was shown as a key participant in cancer cell biology. Feng et al. reported that ASMTL-AS1 was significantly downregulated in papillary thyroid carcinoma, which served as a tumor suppressor via inhibiting cell growth and glycolysis[13]. However, another study showed that ASMTL-AS1 was an oncogene in hepatocellular carcinoma that activated carcinogenic YAP signaling and promoted cancer recurrence or metastasis[14]. Herein, we found that ASMTL-AS1 was a tumor-inhibiting lncRNA in TNBC, inactivating oncogenic Wnt/β-catenin pathway and repressing cell proliferation and invasion, and restoration of ASMTL-AS1 expression significantly retarded the growth of tumor *in vivo*. The discrepancy of ASMTL-AS1 function in different cancer types can be explained by the cell type- or developmental stage-specific functional pattern of lncRNA[25]. The expression level and functional mechanism of ASMTL-AS1 in other malignant tumors need to be further studied.

## Conclusions

Overall, our findings reveal the previously uncharacterized anti-tumor effect of ASMTL-AS1 in TNBC, in which ASMTL-AS1 acts as a ceRNA sponging miR-1228-3p and elevating SOX17, resulting in the inactivation of Wnt/β-catenin signaling, thereby inhibiting TNBC tumorigenesis and progression. Hence, we provide a potential prognostic indicator and therapeutic target for TNBC patients.

## Data Availability

The data sets used and/or analyzed during the current study are available from the corresponding author on reasonable request.

## Abbreviations

TNBC: Triple-negative breast cancer
LncRNA: long-non coding RNA
qRT-PCR: Quantitative real-time polymerase chain reaction
miRNA: microRNA
ceRNA: Competing endogenous RNA
